# Supplementary material for: Structure and Properties of Sprayed Polyurethane Bio-Based Foams Produced Under Varying Fabrication Parameters
Source: Polymers (Basel). 2025 Sep 18;17(18):2522. doi: 10.3390/polym17182522 (PMC12473794; doi:10.3390/polym17182522)
Supplement: Supplementary file 1 [file polymers-17-02522-s001.zip › polymers-3825610-supplementary.pdf]

S1. Description of the image analysis procedure

Statistical algorithms were used to determine pores distribution and dimensions. An example of analysis is shown in Fig S1

There are following steps:

1. The batch folder was opened (upper left);
2. A dedicated macro was used to delineate pore borders (upper right);
3. Particle analysis was performed to determine the number and area of pores (lower right);
4. A mask was merged with the original image (lower left).

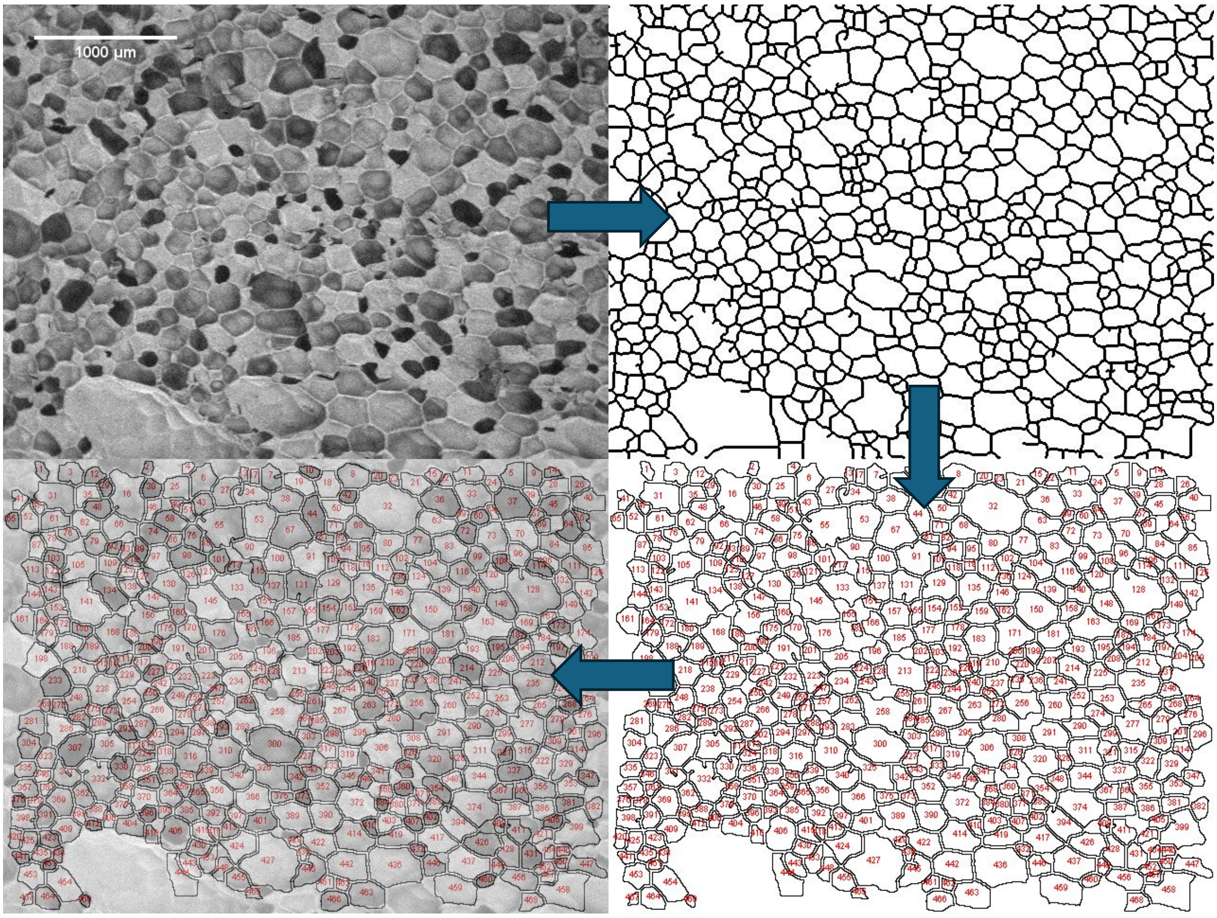

Figure S1 1 An example of SEM analysis

Each foam sample was represented by 15 SEM micrographs taken at 40× magnification, with each image containing approximately 400–500 pores. This provided a statistical base of over 7,000 measurements per foam type. The images were taken both perpendicular and parallel to the growth direction.

Microscopy images were processed using ImageJ software, employing a defined image analysis workflow. Custom macros—a sequence of commands automating image processing—were developed in ImageJ. Subsequently, particle analysis was conducted, yielding statistical distributions from a minimum of 3,000 pores per image series. To reduce error from non-pore

artifacts, objects smaller than 20 μm were excluded. Statistical averages were then computed. Additionally, pore density per unit area was analyzed using the planimetric method, commonly used in grain analysis [1], [2].

Macro

Table S1 presents the macro employed in ImageJ to process microscope images. The macro generates a data summary suitable for further statistical analysis.

Table S1: ImageJ macro used for calculations

```
//Opening the whole folder of pictures with proper names

//setting the scale according to picture dimensions
run("Set Scale...", "distance=302 known=2000 unit=um global");


//Cutting the bottom part, which is not expected
makeRectangle(0, 0, 1280, 960);
run("Crop");

//First step of finding the edges
run("Find Edges", "stack");

//making the image binary
setAutoThreshold("Default");
run("Convert to Mask", "method=Default background=Light calculate black
stack");

//image further modification, to achieve good pore closing
run("Remove Outliers...", "radius=3 threshold=50 which=Bright stack");
run("Despeckle", "stack");
run("Median...", "radius=2 stack");
run("Watershed", "stack");
run("Minimum...", "radius=2 stack");
run("Maximum...", "radius=2 stack");
run("Invert", "stack");
run("Skeletonize", "stack");
run("Invert", "stack");
run("Minimum...", "radius=0.2 stack");

//particle analysis
run("Analyze Particles...", "size=4000-Infinity circularity=0.250-1.00
show=Outlines display exclude clear stack");
```

S2. Supplementary data for DSC analysis

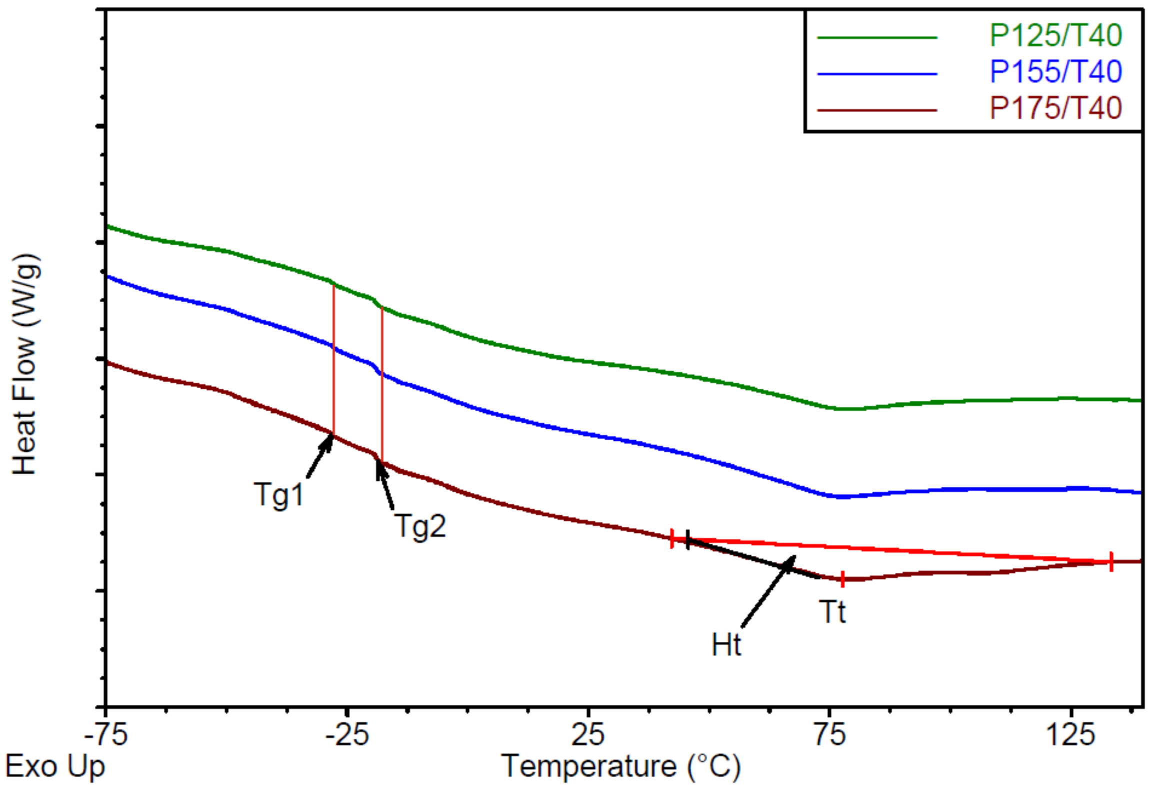

Figure S2 DSC thermograms of foams made at varying mixing pressures and a constant substrate temperature of 40°C

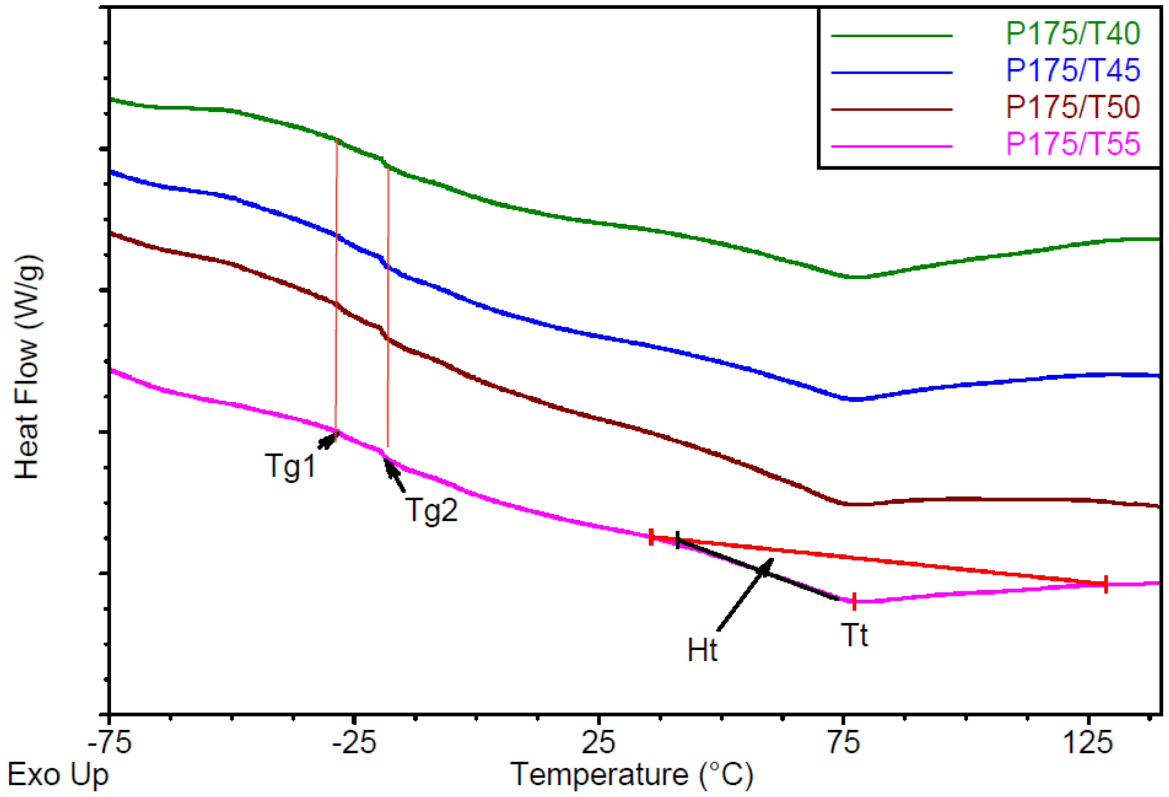

Figure S3 DSC thermograms of foams made at a constant mixing pressure of 175 bar and varying substrate temperature

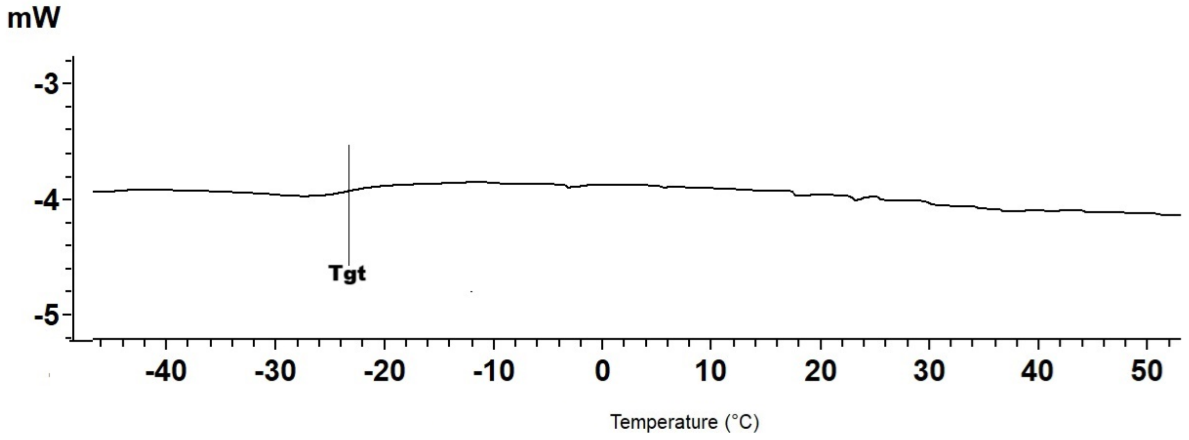

Figure S4 DSC thermogram of tall oil polyol

S3. Supplementary data for FTIR analysis

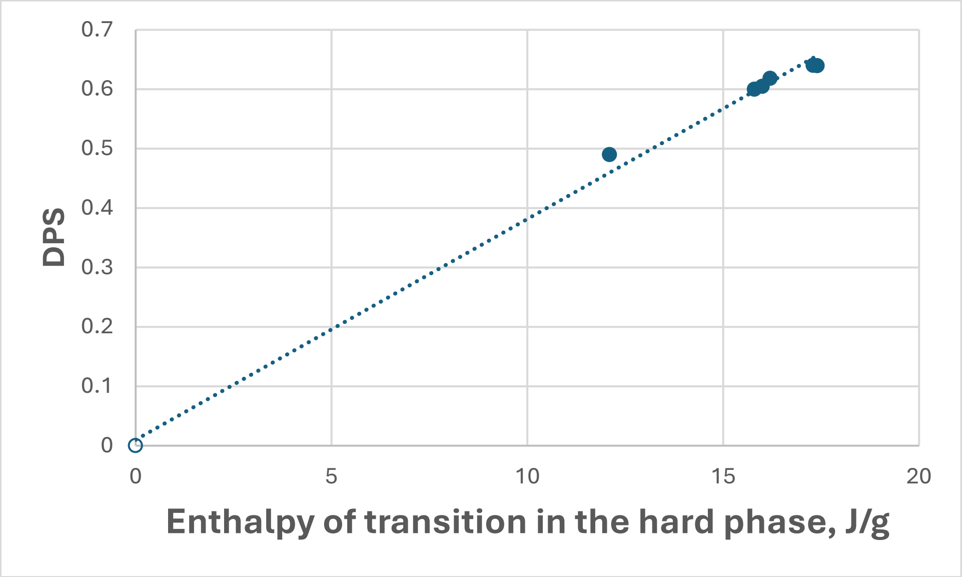

Figure S5 5 Dependence of the change in transition enthalpy in the hard phase of the tested foams and the degree of phase separation of their hard phase

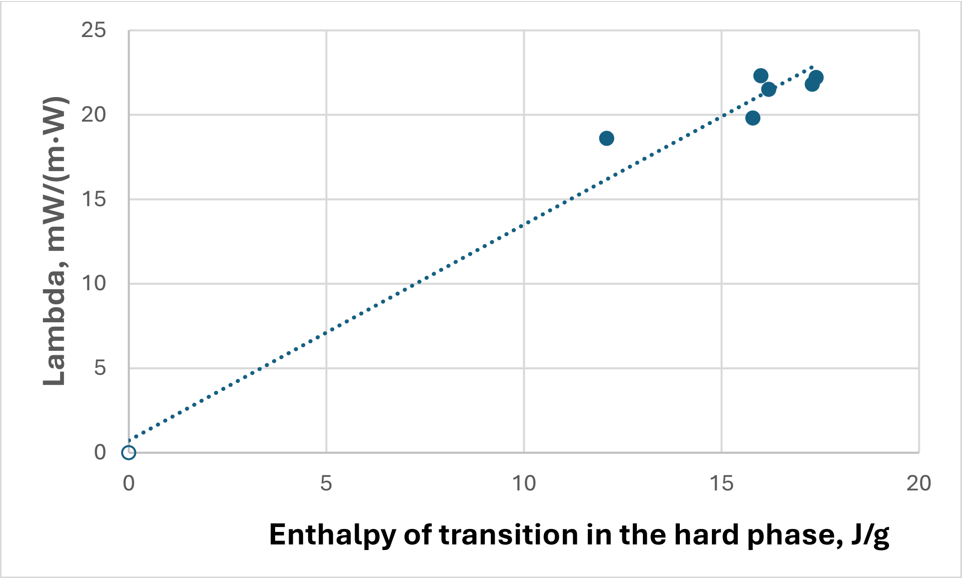

Figure S6 Dependence of the change in transition enthalpy in the hard phase of the tested foams and the thermal conductivity coefficient of the foams tested

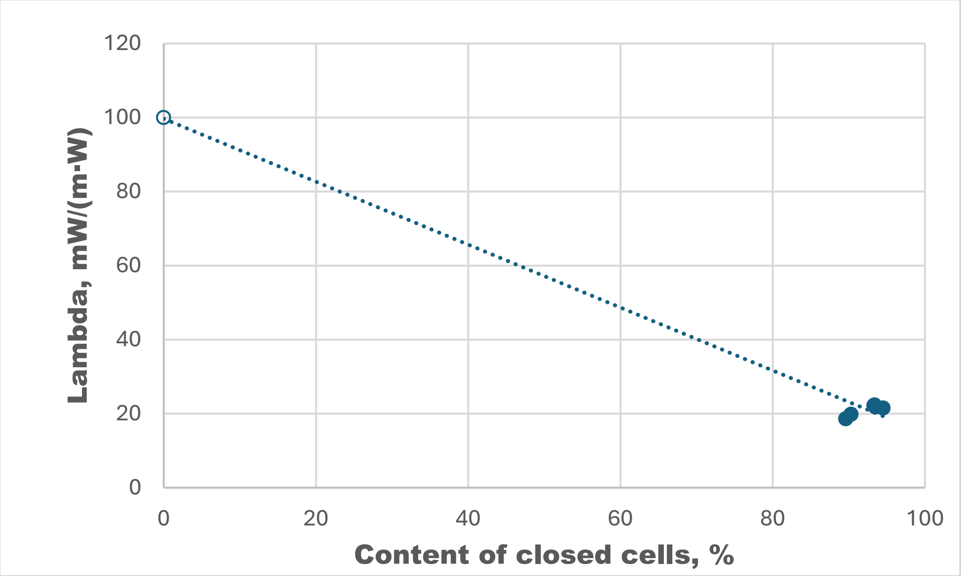

Figure S7: Dependence of closed cell content and thermal conductivity coefficient of the tested foams. Assumed to be 100 mW/(m-W) for solid polyurethanes [3]

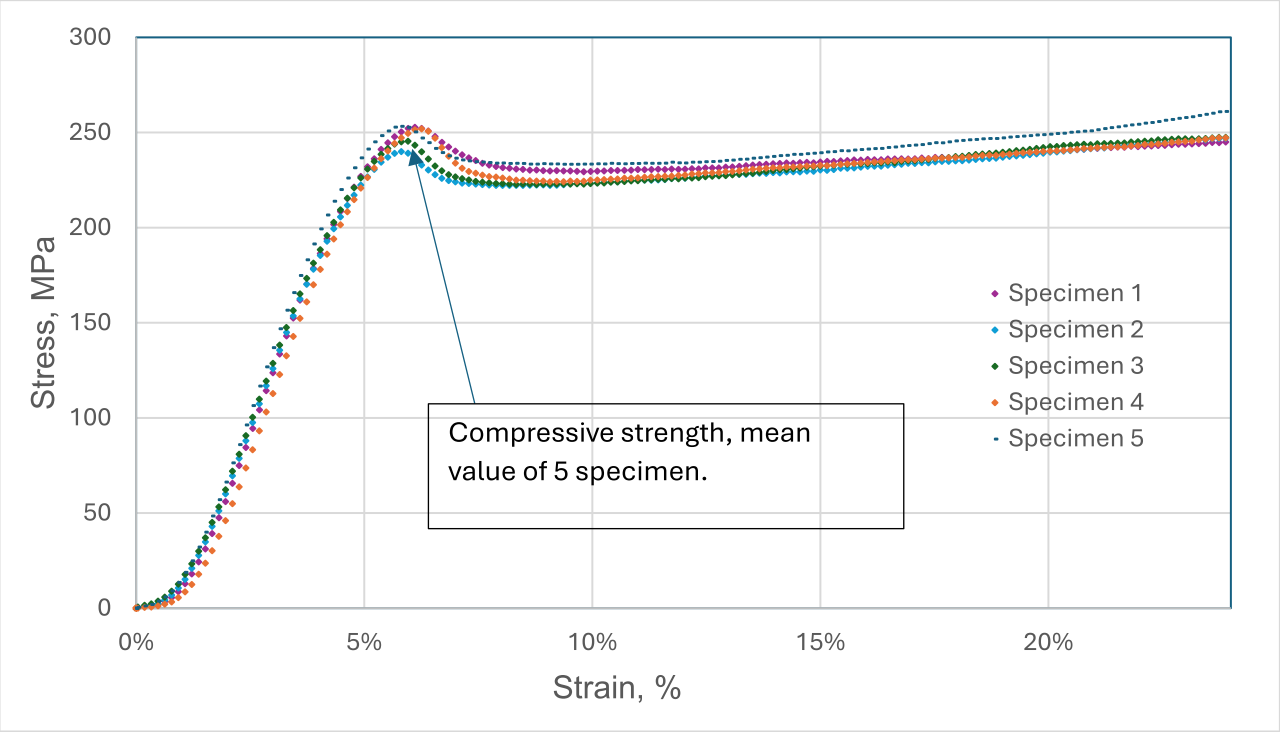

Figure S8: An example of stress-strain curve.

[1] Kurzydłowski, K.J. Praktyka analizy obrazu; Wydawnictwa Naukowo-Techniczne: Warszawa, 1995.

[2] Jastrzębska, I.; Piwowarczyk, A. Traditional vs. Automated Computer Image Analysis—A Comparative Assessment of Use for Analysis of Digital SEM Images of High-Temperature Ceramic Material. Materials 2023, 16, 812

[3] Gallagher Corporation. *Physical Constants of Urethane Elastomers*. Available online: https://gallaghercorp.com/physical-constants-urethane-elastomers/ (accessed on 17 April 2025).
